# Household behavioural responses following successful IRS malaria control: challenges for health education and intervention strategies

**DOI:** 10.1186/1475-2875-11-S1-P12

**Published:** 2012-10-15

**Authors:** Maria (Riana) Bornman, Henrik Kylin, Hindrik Bouwman

**Affiliations:** 1 Department of Urology and University of Pretoria Centre for Sustainable Malaria Control, University of Pretoria, Pretoria 0001, South Africa; 2 Department of Water and Environmental Studies, Linköping University, 58183 Linköping, Sweden; 3 School of Environmental Sciences and Development, North-West University, Potchefstroom 2520, South Africa

## Background

Control of malaria remains one of the world’s chief current public health challenges, particularly in sub-Saharan Africa [1] where malaria is still responsible for 10% of the total disease burden. Mothers, guardians and caregivers of children play a vital role in the prevention, early detection and management of malaria. The general and daily priorities of caregivers living in a malarial area are not well understood, particularly as they have to balance competing social, economic and health constraints. A better understanding of household behaviour with respect to health education is imperative for the reduction of malaria incidence and the success of malaria control strategies.

The investigation compared the relative importance assigned by female caregivers in communities under a successful vertically-managed malaria control programme to malaria awareness on the one hand and to social and economic concerns on the other.

## Materials and methods

We conducted interviews with 156 caregivers of children using both open-ended and closed fixed-answer questions. The adult female responsible for the day-to-day care of the children was interviewed 1) in two malarial villages subject to annual indoor residual spraying (IRS) a total of 120 persons (60 in each village), and 2) 36 in a reference non-sprayed village.

## Results

The mean income was between 27-56% of the national mean, indicating a community under considerable pressure. Male parents were often absent due to work commitments. Unemployment, poverty, crime, and lack of clean water were the main, unprompted, threats, but malaria was volunteered by none. Only when malaria was prompted (caregivers had good knowledge of malaria), did its concern rise to 52% and 38% in the IRS-sprayed villages.

## Conclusions

Malaria was not a prominent conscious concern and this apparent discrepancy between actual daily and potential future threats significantly increases the difficulty of mobilising communities for preventive action regarding potential threats. Integrated Vector Management (IVM) [2], a multi-sector (central or local government, together with communities) horizontal control program, may be particularly difficult to implement in communities such as these above. Any changes to this effective system will therefore have to take exceptional care not to impact on the effectiveness other than to improve it. Considerations should be given to study the needs requirements of the caregiver as a crucial component of rural community life as their ‘buy-in’ to any new measures will be crucial for success. Our findings should be considered in malaria control strategies, rural policy development, climate change adaptation, and communication strategies [3].
